# Quantitative measure of concrete fragment using ANN to consider uncertainties under impact loading

**DOI:** 10.1038/s41598-022-15253-z

**Published:** 2022-07-04

**Authors:** Kyeongjin Kim, WooSeok Kim, Junwon Seo, Yoseok Jeong, Jaeha Lee

**Affiliations:** 1 Major of Civil Engineering, National Korea Maritime & Ocean University, Busan, 49112 Republic of Korea; 2 Interdisciplinary Major of Ocean Renewable Energy Engineering, National Korea Maritime & Ocean University, Busan, 49112 Republic of Korea; 3 Department of Civil Engineering, Chungnam National University, 99 Daehak-ro, Yuseong-gu, Daejeon, 34134 Republic of Korea; 4 Department of Civil and Environmental Engineering, South Dakota State University, Brookings, SD 57007 USA; 5 Department of Construction and Disaster Prevention Engineering, Kyungpook National University, 2559 Kyungsangdae-ro, Sangju, 37224 Republic of Korea

**Keywords:** Engineering, Civil engineering

## Abstract

In this study, numerical analysis was performed to predict amount of fragments and travel distance after collision of a concrete median barrier with a truck under impact loading using Smooth Particle Hydrodynamics (SPH). The obtained results of the SPH analysis showed that amount of fragments and the travel distance can be changed depending on different velocity-to-mass ratios under same local impact energy. Using the results of the SPH analysis, artificial neural network (ANN) was constructed to consider the uncertainties for the prediction of amount of fragments and travel distance of concrete after collision. In addition, the results of the ANN were compared with the results of multiple linear regression analysis (MRA). The ANN results showed better coefficient of determination (R^2^) than the MRA results. Therefore, the ANN showed improvement than the MRA results in terms of the uncertainties of the prediction of amount of fragments and travel distance. Using the constructed ANN, data augmentation was conducted from a limited number of analysis data using a statistical distribution method. Finally, the fragility curves of the concrete median barrier were suggested to estimate the probability of exceed specific amount of fragments and travel distance under same impact energy.

## Introduction

**Figure 1:**
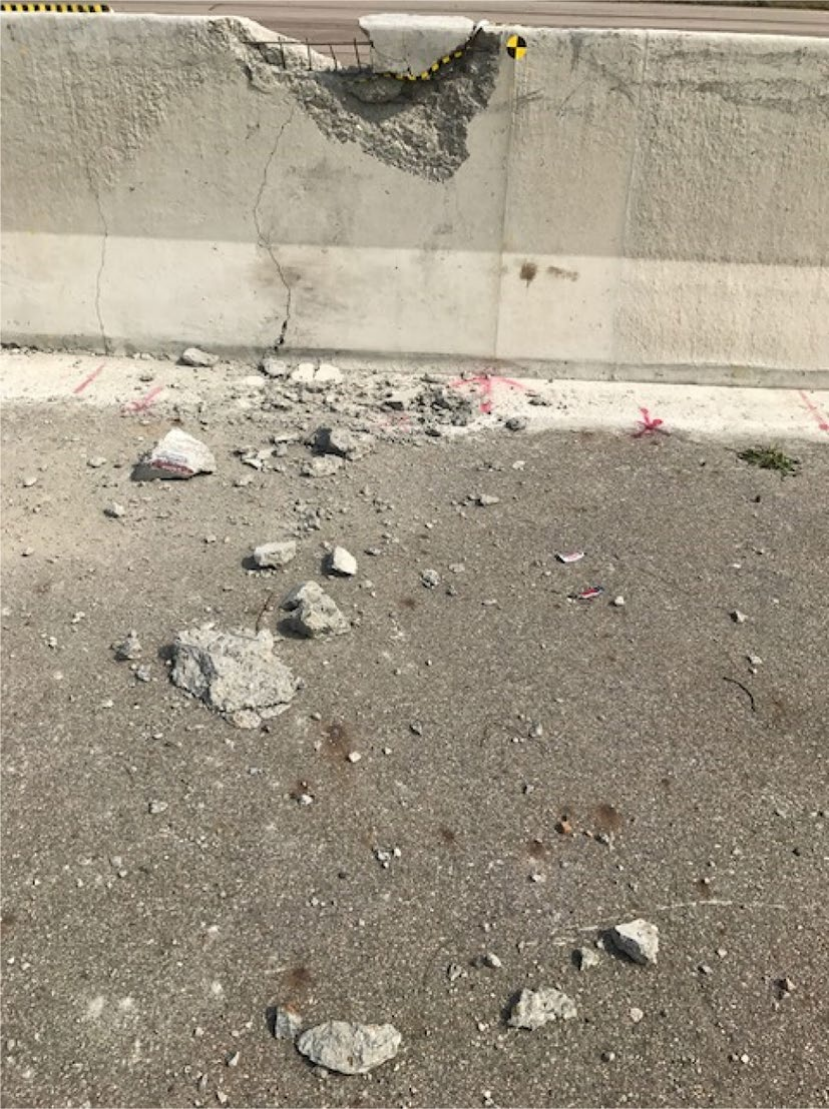
Concrete fragments due to CMB-truck collision.

Concrete median barriers (CMB) on highways are typical road safety facilities which often undergo breakage during truck collisions, as shown in Fig. 1. The concrete fragments generated from the broken CMBs during such collisions occur severe threats because they fly around and hit other approaching trucks in the opposite direction, thereby causing secondary accidents. Therefore, the prediction of amount of fragments and travel distance depending on the specification of the cross-section and impact severity is imperative to prevent further secondary accidents. The generated amount of fragments and the travel distance require accurate predictions. However, there are always uncertainties in such predictions due to strain rate effect, size effect, heterogeneous material and characteristics of the impactor.

If the prediction of amount of fragments and travel distance according to the collision conditions can be improved to a reasonable level, the design process of the CMBs can be tailored likewise, which in turn can reduce further fragment-related accidents.

In South Korea, according to real impact test guideline for vehicle safety guard 2015, the CMBs are considered to lack suitable performance if there are more than 2 kg of fragments and 2 m of the travel distance^1^. However, such guidelines are only followed in the evaluation of the field test and not followed in the design and analytical process of the CMBs.

To note, it is not easy to predict the amount of fragments and there is always some uncertainties in the travel distance because prediction of the amount of fragments under impact loading shows sensitive results depending on the specifications of the cross-section and the impactor. In particular, if there is any difference in amount of fragments due to combination of impact velocity and impact mass on the same local impact energy, it may be necessary to improve the standard established.

In the past, to predict the behavior of concrete under an impact load, finite element method (FEM) was adopted by several researchers, such as Orbovic et al.^2^, Zhang et al.^3^, Ranade et al.^4^, Kim et al.^5^ and Kim et al.^6^. However, according to Lee et al.^7^, Lee et al.^8^ and Kim et al.^9^, the FEM has certain limitations in predicting amount of fragments and travel distance since it simulates fragments through element deletion.

Therefore, in place of FEM, Smooth Particle Hydrodynamics (SPH), which is mesh-free method by Rabczuk et al.^10^, can be used to predict amount of fragments and travel distance of the fragments under impact load.

**Figure 2:**
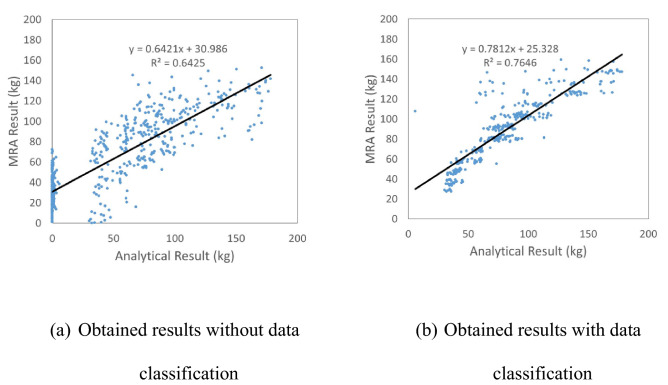
Comparison of analytical results with MRA prediction^11^.

Recently, Kim et al.^11^ used SPH to estimate the amount of fragments. In addition, Multiple Linear Regression Analysis (MRA) was conducted based on concrete thickness, compressive strength, reinforcement ratio, impact velocity, impact mass and impact location. However, the data obtained from MRA did not show high coefficient of determination (R^2^) because in MRA there remains an difference in the linear relationship between the independent and dependent variables with uncertainties, as shown in Fig. 2 regardless of using data classification. Thus, to predict amount of fragments and travel distances with uncertainties, artificial neural network (ANN) was used which considers nonlinearity.

In this study, to evaluate the structural performance of CMB, impact analysis was performed using SPH and analytical results were used for a construction of ANN. Accordingly, amount of fragments and travel distance were predicted using ANN. The results of the ANN were also further compared with the field test. Finally, fragility curves of specific CMB have been presented to evaluate the structural performance, i.e. amount of fragments and travel distance based on ANN.

## The need for development of local impact model

Typically, the truck FE model of National Crash Analysis Center (NCAC), based on the European standard EN-1317, is widely used for truck collision analysis around the world. However, the truck model was originally developed for the global impact analysis so it is not proper to apply to the local impact model.

Therefore, it is necessary to develop a detailed local analysis model which would simulate such a local collision, and define external forces (impact mass and impact velocity).

### Local impact model based on secondary collision

**Figure 3:**
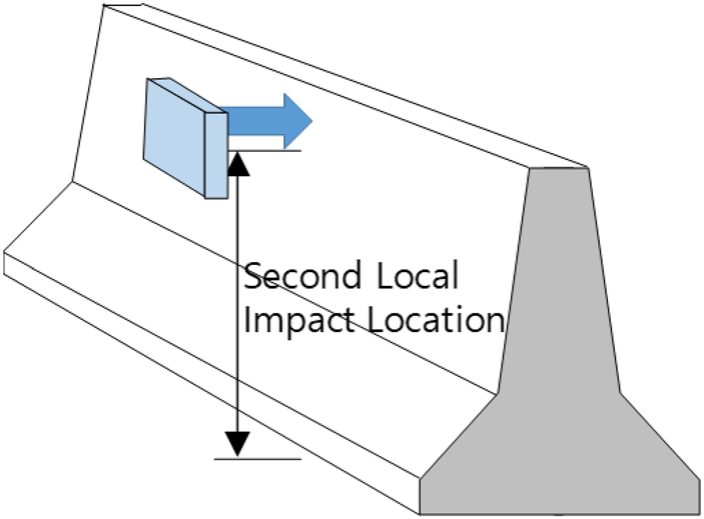
Conceptual drawing of the local impact SPH model.

From the results of the field impact test between a CMB and truck, three major impacts were identified. First, the bumper zone of the truck made contact. Second, the corner of the truck cargo compartment collided with the upper zone of the CMB. The third impact was collision between the CMB and rear wheels of the truck. The largest amount of fragments was generated in secondary collision according to previous studies^9,12,13^. Therefore, a local impact model was developed to predict the largest amount of fragments during secondary collision. Figure 3 shows the concept of the local impact model.

### Research scopes for parametric study

The standard deviation value and average value of the total mass of the truck involved in the concrete median barrier collision were found from the statistical data of the Korea expressway^14^. However, the partial mass of the total truck mass involved in the local impact was unknown. Therefore, reverse analysis was conducted to estimate the partial mass, and consequently, the secondary impact energy could also be found.

The impact location and impact velocity were obtained from the video data of field test^15–17^. To estimate the local impact mass, reverse analysis was conducted and compared with the damaged area and crack patterns after collision. Then, using the ratio of the estimated local impact mass to the total truck mass and range of the local impact velocity obtained from the video data, the range of local impact energy was found to be from minimum value of 3.2 kJ to maximum 18.0 kJ, giving an average value of 10.8 kJ. Detailed information on the estimation of the impact energy can be found in Kim et al.^11^. Accordingly, the scope of impact energy for SPH analysis was selected as shown in Table 1. The scope of compressive strength and thickness of CMB and reinforcement ratio were determined after statistical reviews on current CMBs serviced in Korea and USA. Impact locations from the top surface was determined after visual inspection from the conducted field tests.

**Table 1:** The scope of the parametric study.

			Minimum	Maximum
Compressive strength			25.5 MPa	34.5 MPa
Thickness			150 mm	250 mm
Reinforcement ratio			0.0	0.4
Impact location from the top surface			80 mm	140 mm
Impact energy	3.2 kJ	Velocity	17.0 km/h	22.8 km/h
		Mass	160 kg	280 kg
	10.8 kJ	Velocity	17.0 km/h	36.0 km/h
		Mass	210 kg	970 kg
	18.0 kJ	Velocity	17.0 km/h	36.0 km/h
		Mass	360 kg	1,600 kg

### Selected parameters for local impact model

In the local analysis model, the concrete was composed of SPH particles whereas the wire-mesh was composed of beam elements. The concrete material model and the wire-mesh for numerical analysis can be found from several studies. Recently, an efficient concrete material model that has been reported for multi-scale analysis^18–20^ is expected to be suitable also for the present study. However, built-in material models such as Continuous Surface Cap (CSC) model^12,13^ and Piecewise Linear Plasticity^21^ in LS-Dyna were selected for this study.

The CSC model was developed for road side safety on highways by the Federal Highway Administration (FHWA)^12,13^. Therefore, the CSC model is suitable typically for low velocity scenarios. Furthermore, the CSC model can simulate strain rate, non-linear behavior in tensile and compression. It can also simulate aggregate size affects under softening behavior of the damage formulation. On the other hand, the Piecewise Linear Plasticity model can simulate the strain rate effect using the Cowper–Symonds equation and the values of the selected average plastic strain rate C and coefficient of the strain rate p were 1.05 × 10^7^ and 8.3, respectively, as reported by Chung et al.^22^.

The maximum aggregate size in the CSC model was set to 19 mm. In the FE analysis, the element size selected for the concrete body was 10 mm. In the SPH analyses, particle spacing of the concrete body was 10 mm and the selected smoothing length was 1.2. The selected minimum and maximum scale factors for the smoothing length were 0.2 and 2.0, respectively. The number of particles in the SPH analysis was minimum 198,990 and maximum 331,650 depending upon the concrete thickness. Assuming a perfect bonding between the concrete and wire-mesh, the wire-mesh shared the same concrete particles and was connected between the adjacent nodes of each concrete particle.

### Verification of developed SPH model

The size of the numerical model was 3000 × 1270 × thickness parameter (mm^3^). Typically, the shape of a CMB becomes thicker toward the lower end. However, in the current study, a reduced numerical model was developed to improve the efficiency by assuming that the CMB has a slab shape. This assumption is logical because the lower part of a CMB does not play any role in reducing the amount of fragments. Furthermore, prior reports^8,9,23^ through preliminary study found that when the length of the CMB was 3000 mm or more, the boundary condition of the fixed side did not affect the generation of fragments in the efficient local impact model. Therefore, the length of the CMB used in the current study was 3000 mm and 10 mm particle spacing.

**Figure 4:**
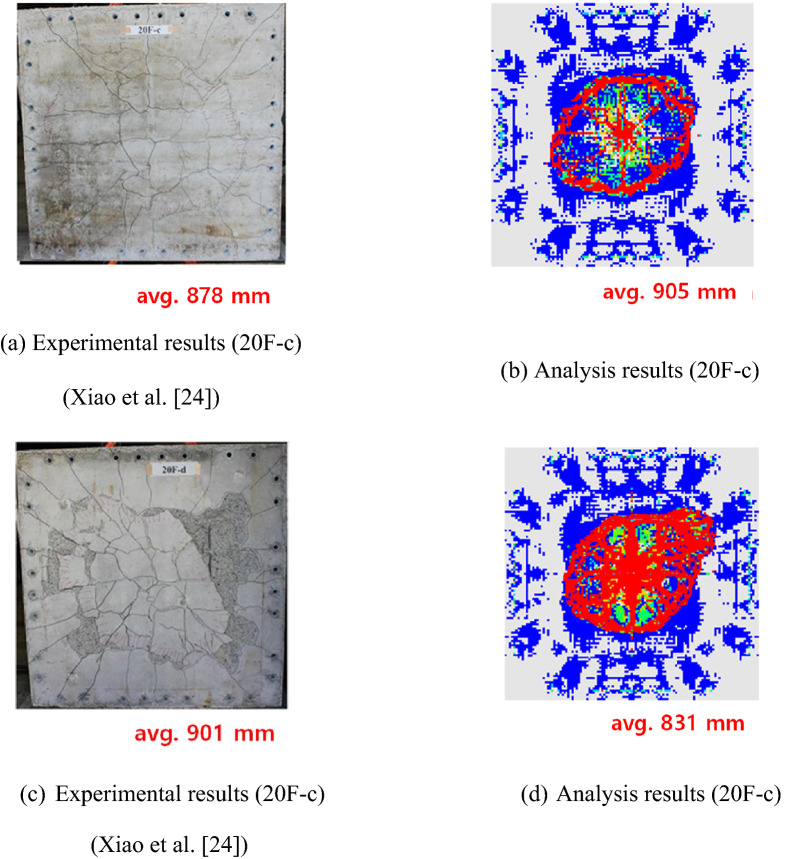
Verification of the SPH numerical model.

Model verification was conducted by comparing with Xiao et al.^24^, since impact velocity, structure size, and shape of projectile are similar to the local impact of a CMB-truck collision. Furthermore, the damaged area and crack patterns of the numerical model were compared with experimental test data, as shown in Fig. 4, and it was concluded that the developed model is acceptable for predicting amount of fragments.

The boundary condition in Xiao’s research was 4 sides fixed condition. However, in the current study, 1 side was kept free and 3 sides fixed since we wanted to estimate the local impact. Similar to Xiao’s research with 4 sides fixed, the results of the current study with 1 side free and 3 sides fixed also showed similar damaged area and crack patterns as those of the field test results. The result indicated that the developed numerical model was suitable for not only 4 sides fixed condition, but also for 1 side free and 3 sides fixed condition to predict the local impact behavior. The comparison results between the developed numerical model and field test can be found in “Comparisons of field test results with SPH analysis, MRA and ANN” section. Furthermore, a preliminary study was conducted to determine the effective length unaffected by boundary conditions. Therefore, the boundary condition for efficient local impact analysis is 1 side free and 3 sides fixed which is a reasonable assumption for efficient local impact analysis. A comparison of detailed verification and development can be found in Kim^25^.

### Prediction of travel distance

**Figure 5:**
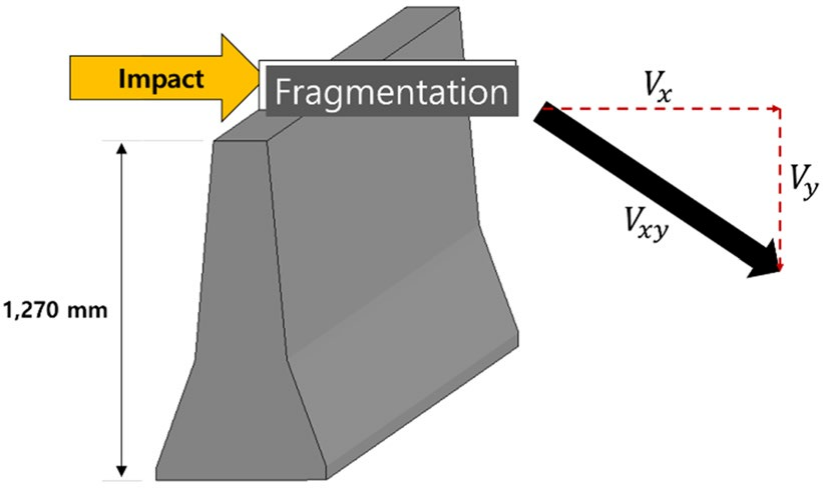
Prediction of travel distance using initial velocity.

The travel distance of fragments was obtained from fully simulated results. However, it was found that to obtain a fully simulated result, it takes more than 150 h with Intel(R) Xeon(R) CPU E5-2643 v3 3.4 GHz. Furthermore, computational convergence and getting accurate results gradually become difficult if flying behavior of the concrete fragments were fully simulated. Therefore, instead, the total travel distance of the fragments was estimated from the velocity of fragments at the moment of separation (normally, 0.06–0.1 s) from the body of the concrete after collision, as shown in Fig. 5. In this study, the velocity was named as initial velocity.

If the initial velocity is ${\mathrm{V}}_{\mathrm{xy}}$, it can be divided into ${\mathrm{V}}_{\mathrm{x}}$ and ${\mathrm{V}}_{\mathrm{y}}$ components. Furthermore, the fall time, i.e. the time taken by the fragments to fall down to the ground, was calculated using the height of the CMB (1270 mm) using Eq. (1).

1 $$1270= {\mathrm{V}}_{\mathrm{y}}\mathrm{t}+ \frac{1}{2}a{t}^{2},$$

where, ${\mathrm{V}}_{\mathrm{y}}$ is the velocity in the direction of gravity (mm/s), t is time (s) and a is gravitational acceleration (9810 mm/s). Therefore, t can be calculated using the quadratic Eq. (1).

The travel distance was predicted using the obtained fall time as follows.

2 $${\mathrm{V}}_{\mathrm{x}}\times \mathrm{t}=\mathrm{L},$$

where, ${\mathrm{V}}_{\mathrm{x}}$ is the lateral velocity (mm/s), t is time (s) and L is the travel distance (mm).

**Figure 6:**
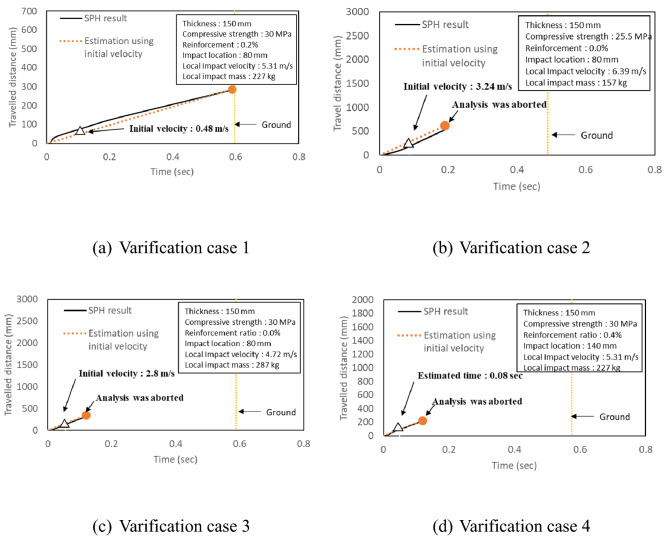
Verification of travel distance of various CMBs.

The estimated travel distance using initial velocity and some fully simulated results were then compared each other as shown in Fig. 6 for the verification. Some analyses were aborted because of convergence problem or computational errors due to excessive distortion in the elements during the simulations. So, the travel distance at the stopped time were compared, as shown in Fig. 6b–d. The difference between the estimated travel distances using initial velocity and fully simulated results were between 2.4 and 7.0%. Considering the computational cost, the travel distance was estimated using the velocity at the moment of separation (initial velocity) when the fragments were generated after collision.

## Application of ANN to evaluate CMBs under local impact

To predict amount of fragments and travel distance of CMB, Kim et al.^11^ performed numerical analysis using SPH and predicted amount of fragments and travel distance using multiple linear regression analysis (MRA). However, the MRA, which linearly defines the relationship between the independent and dependent variables, did not show a high coefficient of determination (R^2^) when uncertainties were considered under impact loadings. Therefore, a method that can define non-linearity, such as ANN was attempted in this study. Kim et al.^8^ compared the amount of fragments and travel distance using ANN and Gradient Boosting Machine (GBM). The ANN was relatively more accurate in predicting the amount of fragments and travel distance after impact. Based on the data obtained from Kim et al.^8^, the current study constructed an improved ANN by conducting a variable study on the key parameters, viz. learning rate, number of layers, epochs and number of nodes. In this regard, more results from the SPH analyses were added, i.e. 12% more training data were used than those used by Kim et al.^25^. Moreover, 1008 h (18 h × 56 models) were additionally spent for analysis using 56 more models based on the previously developed Kim’s model^25^. The accuracy of the amount of fragments and travel distance of the CMB under impact loading were improved by using ANN with more training data.

### Development of ANN in machine learning

ANN is a machine learning technique which is consist of input layer, hidden layers and output layer. The neurons (node) have associated weights and exchange information with each other.

**Figure 7:**
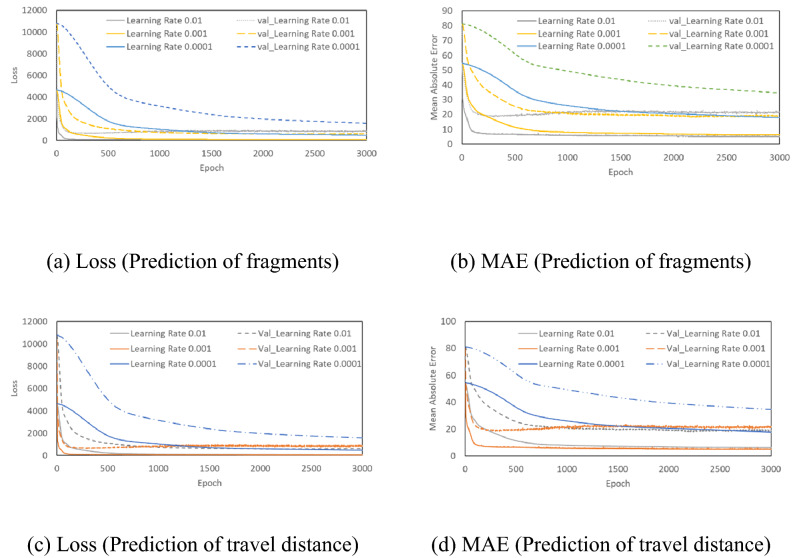
Loss and MAE depending on the learning rate.

It is also important to confirm that overfitting does not occur while minimizing the differences in the ANN learning process. In our study, optimal designs were employed while developing ANN for parametric study of the learning rate, number of layers, epoch, and number of nodes. The loss and mean absolute error (MAE) depending on epoch and learning rate are shown in Fig. 7. The results of train data and validation data showed that learning progressed rapidly at a learning rate of 0.01. However, it can cause non-convergence. By contrast, a learning rate of 0.0001 required a lot of epochs to stabilize. Therefore, for efficiency prediction model, learning rate of 0.001 was selected so that the learning progresses stably. It was also found that epochs of value 3000 was appropriate for this study.

**Figure 8:**
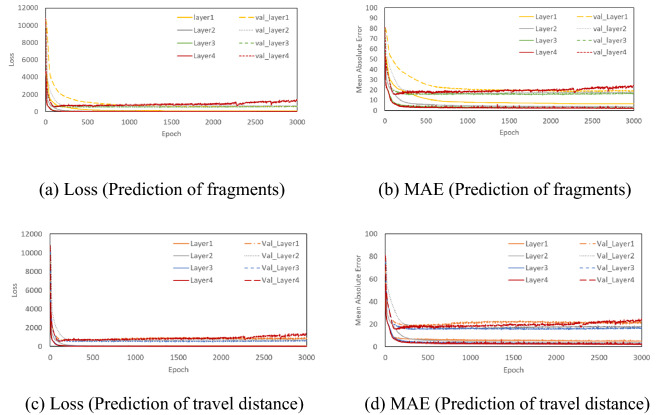
Loss and MAE depending on the number of layers.

The loss and MAE depending on the number of hidden layers are shown in Fig. 8. For 1 hidden layer, the MAE and loss of train data were found to be inappropriate since the loss and MAE were slightly higher than the results of the 2, 3, and 4 hidden layers. In the present study, finally 4 hidden layers were selected after taking into account the results from all variables, such as learning rate, epoch and the number of nodes.

**Figure 9:**
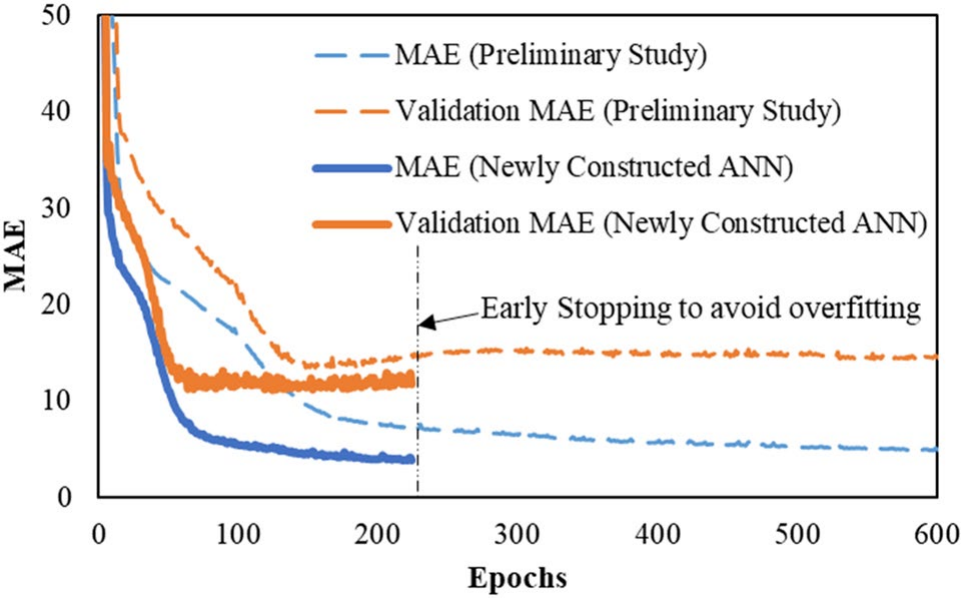
Comparison of the improved performance of the constructed ANN.

The number of nodes did not have any fixed value. In general, the number was set by referring to the number of bits of PC, such as 8 bit, 16 bit, 32 bit, 64 bit, and 128 bit. Atici et al.^26^ recommended that the required number of nodes in the 1st hidden layers should be more than twice the number of the input parameters. At the same time, the number of nodes in the final hidden layer (i.e. the 4th hidden layer in our study) should be reduced to avoid overfitting with low MAE and loss. Therefore, the number of nodes from the 1st to 4th hidden layers used were 64, 32, 16 and 8, respectively for the 6 input parameters (thickness, collision location, compressive strength, reinforcement ratio, impact mass, impact speed). Finally, learning rate of 0.001, epochs 3000, 4 hidden layers and 64, 32, 16 and 8 nodes in each of the 4 hidden layers were selected for the ANN. The selected optimizer and activation function used were Adam and Rectified Linear Unit (ReLU) which were efficient and can avoid the vanishing gradient problem. A detailed discussion on ReLU along with an example can be found in Hinton^27^, Nair and Hinton^28^ and Glorot et al.^29^. The current research used ANN constructed by enhancing the previous ANN built by Kim et al.^8^. To predict the amount of fragments and travel distance of concrete, an optimized ANN was built and used in a variable study for performance improvement of ANN based on ReLU, Stochastic Gradient Descent, Adam optimizer. Table 2 shows the selected key parameters compared to those of Kim et al.^8^, whereas Fig. 9 shows the constructed ANN with those selected parameters.

**Table 2:** Selected key parameters.

Factor	Selected parameter (Kim et al.^8^)	Selected parameter in this study
Epoch	2000	3000
Learning rate	0.001	0.001
Number of hidden layer	3	4
Number of node	32, 16, 8	64, 32, 16, 8
Number of training data	486	542
Early stopping	No	Yes

### Obtained results from the constructed ANN

**Figure 10:**
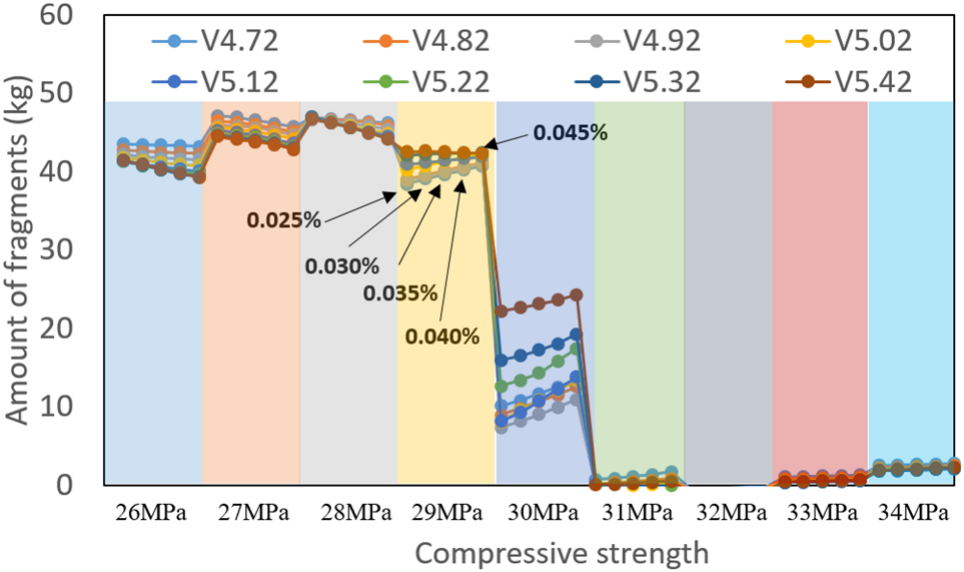
Amount of fragments depending upon various local impact velocities under same local impact energy.

Figure 10 shows the ANN prediction on the amount of fragments depending on various impact velocities under same impact energy. For example, V4.17 in Fig. 10 indicates the velocity of 4.72 m/s. For all cases, the thickness of CMB was uniform 150 mm. In Fig. 10, with the same unit of compressive strength, as the bullet moved to the right, the reinforcement ratio increased by 0.05 from 0.025 to 0.045. As expected, the overall results from the ANN showed that the amount of fragments decreased as the compressive strength and reinforcement ratio increased. The obtained results in Fig. 10 also showed the stepwise changes in the amount of fragments at a specific compressive strength. It is thought that it was related to the sensitivity of the ANN learning with limited data. Had more analysis results been continuously added and updated, the sensitivity of the prediction would have improved further.

### Comparison of the MRA and ANN results

**Figure 11:**
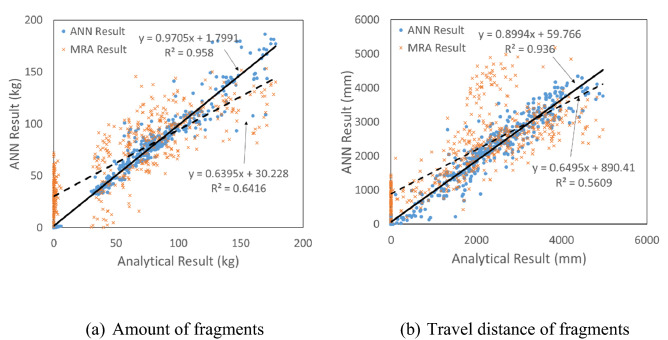
Comparison of the MRA and ANN.

The ANN results of the amount of fragments and travel distance were shown in Fig. 11a,b compared to MRA results which were previously explained. A total of 542 analyses were performed within the scope, as explained in “Research scopes for parametric study” section.

This result indicated that the coefficient of determination of ANN (R^2^ = 0.958) increased to much better value than that of MRA (R^2^ = 0.6416), as shown in Fig. 11a. Therefore, the coefficient of determination from the ANN prediction (R^2^ = 0.958) was increased by 49% when compared with the results of the MRA prediction (R^2^ = 0.6416).

The coefficient of determination (R^2^ = 0.936) of the predicted travel distance also improved when compared with that of the MRA (R^2^ = 0.561), as shown in Fig. 11b. The coefficient of determination of the ANN prediction (R^2^ = 0.936) was increased by 67% when compared with the results of the MRA prediction (R^2^ = 0.5609).

The above results indicated that ANN is more appropriate than MRA in predicting values from various input parameters considering the uncertainties.

**Figure 12:**
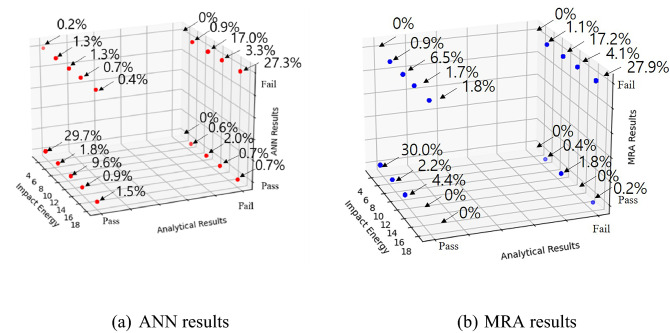
Comparison of the MRA and ANN results regarding fragments and travel distance.

It was confirmed that the newly developed ANN was suitable for detailed prediction of the amount of fragments and the travel distance. By judging this comprehensively, the predicted pass and fail against the Real Impact Test Guideline for Vehicle Safety Guard 2015 (exceed amount of fragments: 2 kg, travel distance: 2 m) suggested by the Korea Expressway^14^ are presented in Fig. 12. The ANN for pass and fail was constructed using logistic regression: It was fail when the value was higher than 0.5 and pass when the value was lower than 0.5. Pass–Pass and Fail–Fail indicated that both analytical and ANN results were identical, while Pass–Fail and Fail–Pass indicated that both results were different. Among the 542 results, the MRA predicted 470 results like the analytical results (86.7%), and the ANN predicted 499 results like the analytical results (92.1%). Therefore, this showed that when the individual prediction performance for the amount of fragments and the travel distance is excellent, the prediction performance to judge comprehensively is also excellent although different regressions were employed.

When the predictions by ANN and analytical results according to the impact energy were different (Pass–Fail, Fail–Pass), the largest error 3.3% (1.3% + 2.0%) of the total occurred at impact energy of 10.8 kN. Therefore, at the impact energy of 10.8 kN, the constructed ANN did not predict the same result as that of the analytical method. This impact energy corresponded to the median value in this study. The uncertainty in prediction became higher since the results for the neighboring values of 7 kN and 14 kN were considered simultaneously.

When the predictions of MRA and analytical results for the impact energy (Pass–Fail, Fail–Pass) were compared, the largest error 8.3% (6.5% + 1.8%) occurred for the impact energy of 10.8 kN, which was similar to ANN. The obtained results indicated that the predictions by ANN are more accurate than the predictions by MRA which predicted linearly. Therefore, the constructed ANN can be used effectively to predict the results before even conducting the real impact tests.

### Comparisons of field test results with SPH analysis, MRA and ANN

A prediction study was performed on SB4 (total impact energy: 160 kJ), which was not included in the ANN train data. The local impact energy of SB4 was estimated (3.6 kJ) from the local impact energy of SB5-B using the energy ratio of SB4 (160 kJ) to SB5-B (270 kJ). The local impact velocity of 18.4 km/h was estimated from the video of the field test, and the partial impact mass 276 kg out of the total truck mass related to local impact was obtained from reverse analysis^17^.

**Figure 13:**
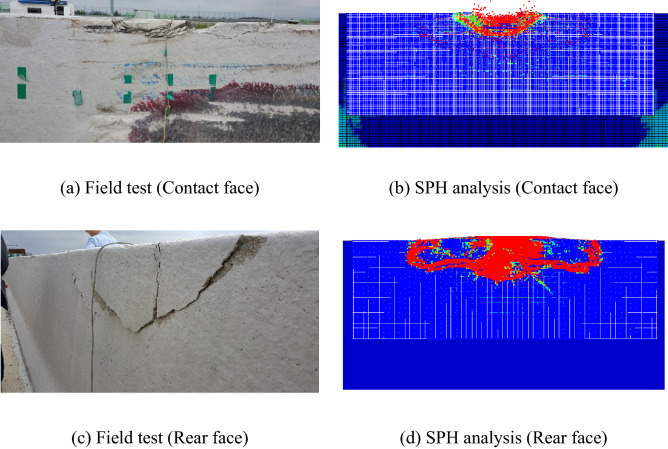
Comparison of field test and SPH analysis.

Figure 13 shows the damaged area of the field test and that of the SPH analysis after collision. The shapes of the damaged area and crack patterns were similar to the field test result, and the spalling behavior at the rear face was properly simulated. Therefore, the developed SPH model can predict the amount of fragments due to spalling.

The prediction results of SPH analysis, MRA and ANN for SB4 (160 kJ) were compared with the results of the field test^23^, as shown in Table 3. The differences between the SPH analysis and the field test results for the amount of fragments and travel distance were 23.5% and 26.9%, respectively. Similarly, the differences between the MRA and the field test results for the amount of fragments and travel distance were 69.2% and 80.8%, respectively. The MRA result showed a larger difference than the results of the SPH analysis. Overall, the best prediction results were obtained from the constructed ANN. The differences between the ANN and the field test for the amount of fragments and travel distance were 7.7% and 36.4%, respectively.

**Table 3:** Results of field test and ANN.

	SB4 Impact severity		
	Fragments (kg)	Travel distance (mm)	Pass/fail
Field test	26	660	Pass
SPH result	33	815	Pass
Prediction of MRA	5	1,117	Pass
Prediction of ANN	28	900	Pass

## Fragility assessment of a CMB under SB4 impact severity using the constructed ANN

Shinozuka et al.^30^ originally used the maximum likelihood method to generate empirical fragility curves using seismic response data from the analysis of bridges. A fragility curve can be expressed as lognormal distribution function with two parameters—the median, and the standard deviation. The estimation of these two parameters is performed via the maximum likelihood method. The well-known likelihood function for the fragility curve can be expressed as shown in Eq. (3).

3 $$\mathrm{L}= \prod_{i=1}^{N}{\left[F\left({a}_{i}\right)\right]}^{{x}_{i}}{\left[1-F\left({a}_{i}\right)\right]}^{1-{x}_{i}},$$

where $\mathrm{F}({\mathrm{a}}_{\mathrm{i}})$: Probability of a specific damage state, ${\mathrm{a}}_{\mathrm{i}}$: A predefined performance boundary requirement, ${\mathrm{x}}_{\mathrm{i}}$: Binary variable equal to 1 or 0 representing success or failure.

A more reliable fragility assessment can be done with a larger number of available data. Therefore, in this study, data augmentation was performed to expand the number of data when the actual number of data were insufficient. Normally, 100 data points are considered as sufficient number for developing a reliable statistical distribution curve. For example, if we consider that there are 100 points in the normal distribution curve, and set the length of the x-axis for each unit quantity to 1, the height of the curve is proportional to the area. Therefore, total 100 data points can be obtained from the summation of x–y data points and the area can be easily calculated. A detailed explanation of data augmentation can be found in Kim et al.^31^. In order to construct the fragility curve of CMB under the local impact, a lognormal cumulative distribution was used with mean value and standard deviation value. The local impact mass or local impact velocity were used as input values instead of peak ground acceleration (a_i_) in maximum likelihood equation by Shinozuka et al.^30^. The next step was to determine whether the approach was successful or not. For example, less than 2 kg of fragments, which was a failure criteria as explained previously, is considered as success. The posterior fragility curve was presented using the mean and standard deviation values which were obtained from the lognormal distribution and calculated from the Bayesian prior distribution.

### The acceptance criteria for an evaluating CMBs

The guidelines of the impact severity for the CMBs in Korea are shown in Table 4^1^. In Korea a certain type of newly developed CMB can be constructed only if it passes the field tests. Two types of field tests were conducted for an evaluation of a CMB: structural performance evaluation and occupant protection performance evaluation. In general, the structural performance evaluation is performed with cargo trucks and occupant protection performance evaluation is performed with small vehicles. The acceptance criteria for the structural performance evaluation of CMB are that no fragments heavier than 2 kg would form, and no fragments would travel more than 2 m. The acceptance criteria for occupant protection performance evaluation are that theoretical head impact velocity (THIV) and permanent head damage (PHD) should be below 33 km/h and 20 g (g: 9.8 m/s^2^) respectively. In this study, using the constructed ANN, only the structural performance evaluation could be considered. Accordingly, a cross-section of CMB designed for SB4 severity was selected and large number of results had been populated using the constructed ANN in order to present fragility curves under the SB4 impact severity. Specific deign used for SB4 CMB could be found from Kim^25^.

**Table 4:** The level of impact severity applied to CMBs in Korea^1^.

Level	Impact velocity (km/h)	Impact mass (kg)	Impact angle (°)	Impact severity (kJ)
SB1	55	8000	15	60
SB2	65			90
SB3	80			130
SB3-B	85			150
SB4	65	14,000		160
SB5	80			230
SB5-B	85			270
SB6	80	25,000		420
SB7		36,000		600

### Fragility curves based on the mass criteria 2 kg for SB4 CMB

**Figure 14:**
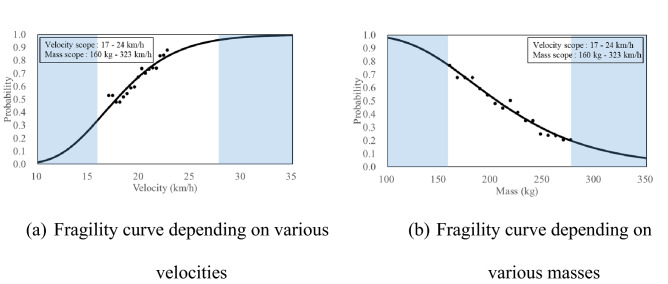
Presented fragility curves under same local impact energy 3.6 kJ (Mass).

Figure 14 shows the fragility curves based on the mass criteria 2 kg under same local impact energy (SB4, total energy:150 kJ, local impact energy: 3.6 kJ) using the SB4 cross-section. The shaded areas in the graph are those that exceed the range of the impact velocity or impact mass explained in Table 1. The 100 mm or 80 mm from the top of the CMBs, obtained from the video data of the field test, were selected as local impact locations, and it was assumed that they all occurred with the same probability. If more field test data could have been accumulated for the impact locations, a more detailed fragility curve could be presented.

Figure 14a,b show the fragility curves depending on the impact velocity and impact mass, respectively, under same local impact energy. In this study, the reason for confirming the fragility curves depending on various velocities and masses while maintaining the same local impact energy condition is that the SPH analysis results in the previous study^11^ showed a certain tendency. Even though the local impact energy was kept same, the increased local impact velocity and mass had effects on the increase and decrease in the amount of fragments, respectively. This tendency was also confirmed by the fragility curves shown in Fig. 14.

The probability of fragments heavier than 2 kg when the local impact velocity of 20.6 km/h was 72% (Fig. 14a), whereas the probability of fragments heavier than 2 kg when local impact mass of 200 kg was 53% (Fig. 14b). The median values, obtained from the fragility curves, for local impact velocity and impact mass were 17.73 km/h (Fig. 14a) and 206 kg (Fig. 14b), respectively. Furthermore, the standard deviation values were 0.26 km/h and 350 kg, respectively.

### Fragility curves based on a criteria of travel distance, 2 m for SB4 CMB

**Figure 15:**
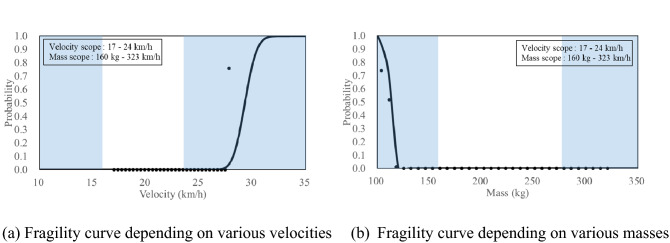
Presented fragility curves under same local impact energy 3.6 kJ (Travel distance).

Figure 15 shows the fragility curves based on the travel distance of 2 m under same local impact energy (SB4, total energy:150 kJ, local impact energy: 3.6 kJ) using the SB4 cross-section. Figure 15a,b show that the fragility curves depending on the impact velocity and impact mass, respectively, on the same local impact energy. The probability of occurrence for the travel distance further than 2 m as the impact velocity increased and the impact mass decreased on the same local impact energy was found. The probability of occurrence for the fragments travelling further than 2 m at 20.6 km/h (Fig. 13a) and 200 kg (Fig. 15b) were both 0%. Therefore, it can be predicted before a field test that a travel distance of the cross section further than 2 m would not occur under SB4 impact energy, and the result of the field test also showed less than 2 m. The detailed results of the field test were previously explained in “Comparisons of field test results with SPH analysis, MRA and ANN” section. The median values, obtained from the fragility curves, for the impact velocity and impact mass were 29.29 km/h (Fig. 15a) and 112 kg (Fig. 15b), respectively. Furthermore, the standard deviation values were 0.03 km/h and 27 kg, respectively. When compared with the results of the fragility curve of the amount of fragments, the fragility curve for occurrence of travel distance further than 2 m showed higher median values and smaller standard deviation values.

Fragility analysis of the CMB designed for SB4 impact severity using the constructed ANN revealed that the probability of the amount of fragments heavier than 2 kg was relatively high, but the probability of travel distance of fragments larger than 2 m was low.

### Fragility curves based on both mass and travel distance criteria for SB4 CMB

**Figure 16:**
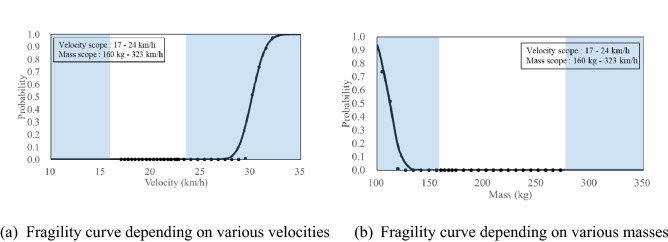
Presented fragility curves under same local impact energy 3.6 kJ (Mass and Travel distance).

Figure 16 shows the fragility curves after accepting both the amount of fragments and travel distance criteria. For amount of fragments and travel distance, the median values were 30.11 km/h (Fig. 16a) and 112 kg (Fig. 16b), and the standard deviation values were 0.04 km/h and 73 kg, respectively. Such a fragility curve can be immediately used to assess the change in the probability of exceedance for design purposes. For example, if the impact velocity is 20.6 km/h, the probability of exceeding the structural performance criteria (more than 2 kg, 2 m) was 0.00, as shown in Fig. 16a. Likewise, if the impact mass 200 kg, the probability of exceeding the structural performance criteria (more than 2 kg, 2 m) was 0.00, as shown in Fig. 16b. It is expected that the probabilities shown in Fig. 16 are much smaller values than those in Fig. 14 since the number of criteria has been increased from one to two and the probability of occurrence for the travel distance further than 2 m was originally 0%, as explained in Fig. 15.

Actually, Fig. 13 and Table 3 showed that in the field test, the CMB designed for SB4 passed in acceptance criteria since the travel distance was less than 2 m while the amount of fragments was much larger than the criteria. Of course, it should be evaluated by applying more field test results. However, the presented fragility curves showed that ANN can be successfully applied to evaluate the structural performance, such as the impact resistance of the CMB under SB4 impact severity with limited number of field test and analysis data.

### Fragility curves based on pass or fail

**Figure 17:**
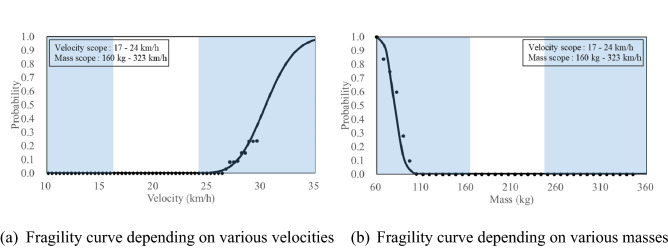
Fragility curves under same local impact energy 3.6 kJ.

Figure 17 shows the probability of failure for the structural performance criteria (more than 2 kg, 2 m) as fragility curves. The presented fragility curve as a function of velocity showed that the median and standard deviation values were 30.43 km/h and 0.07 km/h. The presented fragility curve as a function of mass showed that the median and standard deviation values were 79.9 kg and 99 kg. The proposed cross-section within the scope (impact energy: 3.6 kJ) of this study was predicted to pass the criteria according to the ANN prediction. In a real test, it satisfied the criterion. The results of the impact tests have been shown in Fig. 13 and Table 3. The ANN, constructed in this study, could be used to predict the result of the collision between vehicles and concrete median barriers. Therefore, it can be used as a means to predict the results of real impact tests, which costs a lot of money and time, by using the cross-sectional data.

## Conclusions and future study

In the present study, to evaluate the structural performance of CMB, impact analysis was performed using SPH. The analytical results were used to build ANN. Finally, fragility curves of specific CMB were obtained to evaluate the structural performance.

- In place of FEM, SPH, which is a mesh-free method was developed with 1 side free and 3 sides fixed condition to predict the amount and travel distance of fragments properly. From developed SPH model, the amount and travel distance of fragments under local impact were successfully verified and estimated.
- Local impact energy was found to be in the range 3.2–18.0 kJ from reverse analysis. 542 SPH models with different combinations of thickness of CMB, compressive strengths of concrete, reinforcement ratios, impact locations, local impact velocities and local impact masses within the scope of research were simulated.
- ANN was constructed using 542 SPH analysis results to predict the amount of fragments and travel distance with uncertainties. It was found that ANN is more appropriate than MRA in predicting values from various input parameters taking the uncertainties into account.
- Using the constructed ANN, fragility curves based on the acceptance criteria for selected CMB (SB4) were found. For example, the probability of fragments heavier than 2 kg and further than 2 m when the local impact velocity of 20.6 km/h were 72% and 0%, respectively. These results were also consistent with the field test results.
- Even though the local impact energy was kept unchanged, the increased local impact velocity and masses had effects on the increase and decrease in the probability of fragments occurrence heavier than 2 kg, respectively. The effect of local impact velocities and masses on travel distance of the fragments were found to be insensitive for the selected CMB under SB4 conditions.
- The presented fragility curves signified that ANN can be successfully applied to evaluate the structural performance of CMB under impact considering the uncertainties. As a further study, if more results under various local impact energies can be added to the presently developed ANN, more reliable fragility curves would be obtained.
- The method used in this study can be utilized as a means to predict the results using simple data before a real impact test which costs a lot of money and time. The method could be used in the fields such as vehicle–bridge guardrail collision and atomic power plant–airplane collision.

## Data Availability

The datasets used and/or analysed during the current study available from the corresponding author on reasonable request.

## References

[1] Korea Ministry of Land, Infrastructure and Transport (2015). Real Impact Test Guideline for Vehicle Safety Guard 2015.

[2] Orbovic N, Tarallo F, Rambach JM, Sagals G, Blahoianu A (2015). IRIS_2012 OECD/NEA/CSNI benchmark: Numerical simulations of structural impact. Nucl. Eng. Des..

[3] Zhang MH, Shim VPW, Lu G, Chew CW (2005). Resistance of high-strength concrete to projectile impact. Int. J. Impact Eng..

[4] Ranade R, Li VC, Heard WF, Williams BA (2017). Impact resistance of high strength-high ductility concrete. Cem. Concr. Res..

[5] Kim K, Lee J (2020). Fragility of bridge columns under vehicle impact using risk analysis. Adv. Civil Eng..

[6] Kim K, Cho H, Sohn D, Lee J (2021). The use of expansive chemical agents for concrete demolition: Example of practical design and application. Constr. Build. Mater..

[7] Lee J, Zi G, Lee I, Jeong Y, Kim K, Kim W (2017). Numerical simulation on concrete median barrier for reducing concrete fragment under harsh impact loading of a 25-ton truck. J. Eng. Mater. Technol..

[8] Lee J, Jeong Y, Kim K, Lee I, Kim W (2019). Experimental and numerical investigation of deformable concrete median barrier. Materials.

[9] Kim W, Lee I, Kim K, Jeong Y, Lee J (2019). Evaluation of concrete barriers with novel shock absorbers subjected to impact loading. Arch. Civil Mech. Eng..

[10] Rabczuk T, Eibl J (2003). Simulation of high velocity concrete fragmentation using SPH/MLSPH. Int. J. Numer. Methods Eng..

[11] Kim K, Kim W, Seo J, Jeong Y, Lee J (2022). The amount prediction of concrete fragments after impact using smoothed particle hydrodynamics. Eng. Fail. Anal..

[12] Murray YD (2007). Users Manual for LS-DYNA Concrete Material Model 159. No. FHWA-HRT-05-062.

[13] Murray YD, Abu-Odeh AY, Bligh RP (2007). Evaluation of LS-DYNA Concrete Material Model 159. No. FHWA-HRT-05-063.

[14] *2014 Highway Traffic Volume Data*. (Korea Expressway Corporation, 2015).

[15] *New High-Functioning Concrete Median Barrier with Low Fragmentation in Impact Event Report, Korea Expressway Corporation*. (Korea Expressway Corporation, 2016).

[16] *Development of Optimized Section for SB6 Level Concrete Median Barrier (Final), Korea Expressway Corporation*. (Korea Expressway Corporation, 2018).

[17] *Concrete Median Barrier Performance Test Result Report: SB4*. (Korea Transportation Safety Authority Korea Automobile Testing & Research Institute, 2020).

[18] Sun B, Xu Z (2021). An efficient numerical method for meso-scopic fatigue damage analysis of heterogeneous concrete. Constr. Build. Mater..

[19] Sun B, Liu X, Xu ZD (2022). A multiscale bridging material parameter and damage inversion algorithm from macroscale to mesoscale based on ant colony optimization. J. Eng. Mech..

[20] Sun B (2021). Adaptive multi-scale beam lattice method for competitive trans-scale crack growth simulation of heterogeneous concrete-like materials. Int. J. Fract..

[21] Livermore Software Technology Corporation (2018). LS-DYNA Keyword User’s Manual.

[22] Chung CH, Lee JW, Kim SY, Lee JH (2011). Influencing factors on numerical simulation of crash between RC slab and soft projectile. J. Comput. Struct. Eng. Inst. Korea.

[23] Kim K, Kim W, Seo J, Jeong Y, Lee M, Lee J (2022). Prediction of concrete fragments amount and travel distance under impact loading using deep neural network and gradient boosting method. Materials.

[24] Xiao Y, Li B, Fujikake K (2017). Behavior of reinforced concrete slabs under low-velocity impact. ACI Struct. J..

[25] Kim, K. Application of SPH and ANN for Prediction of the Amount of Concrete Fragmentation under Impact Loadings [dissertation], National Korea Maritime and Ocean University, Busan, South Korea (2021).

[26] Atici U (2011). Prediction of the strength of mineral admixture concrete using multivariable regression analysis and an artificial neural network. Expert Syst. Appl..

[27] Hinton, G. E. Rectified linear units improve restricted Boltzmann machines Vinod nair (2010).

[28] Nair, V. & Hinton, G. E. Rectified linear units improve restricted boltzmann machines*.* In *Icml*. (2010).

[29] Glorot, X., Bordes, A. & Bengio, Y. ReLU. In *AISTATS’11 Proc. 14th Int. Conf. Artif. Intell. Stat.* (2011).

[30] Shinozuka M, Feng MQ, Lee J, Naganuma T (2000). Statistical analysis of fragility curves. J. Eng. Mech..

[31] Kim JHJ, Phan HD, Kim BY, Choi JW, Han TS (2012). Development of satisfaction curves to evaluate concrete mix design performance using a Bayesian probabilistic method. Constr. Build. Mater..

